# An iteration method for identifying yeast essential proteins from heterogeneous network

**DOI:** 10.1186/s12859-019-2930-2

**Published:** 2019-06-24

**Authors:** Bihai Zhao, Yulin Zhao, Xiaoxia Zhang, Zhihong Zhang, Fan Zhang, Lei Wang

**Affiliations:** 1 College of Computer Engineering and Applied Mathematics, Changsha University, Changsha, Hunan 410022 People’s Republic of China; 2 College of Information Engineering, Xiangtan University, Xiangtan, 411105 Hunan China; 3 Hunan Provincial Key Laboratory of Nutrition and Quality Control of Aquatic Animals, Department of Biological and Environmental Engineering, Changsha University, Changsha, Hunan 410022 China

**Keywords:** Heterogeneous network, Protein-protein interaction, Essential proteins

## Abstract

**Background:**

Essential proteins are distinctly important for an organism’s survival and development and crucial to disease analysis and drug design as well. Large-scale protein-protein interaction (PPI) data sets exist in *Saccharomyces cerevisiae*, which provides us with a valuable opportunity to predict identify essential proteins from PPI networks. Many network topology-based computational methods have been designed to detect essential proteins. However, these methods are limited by the completeness of available PPI data. To break out of these restraints, some computational methods have been proposed by integrating PPI networks and multi-source biological data. Despite the progress in the research of multiple data fusion, it is still challenging to improve the prediction accuracy of the computational methods.

**Results:**

In this paper, we design a novel iterative model for essential proteins prediction, named Randomly Walking in the Heterogeneous Network (RWHN). In RWHN, a weighted protein-protein interaction network and a domain-domain association network are constructed according to the original PPI network and the known protein-domain association network, firstly. And then, we establish a new heterogeneous matrix by combining the two constructed networks with the protein-domain association network. Based on the heterogeneous matrix, a transition probability matrix is established by normalized operation. Finally, an improved PageRank algorithm is adopted on the heterogeneous network for essential proteins prediction. In order to eliminate the influence of the false negative, information on orthologous proteins and the subcellular localization information of proteins are integrated to initialize the score vector of proteins. In RWHN, the topology, conservative and functional features of essential proteins are all taken into account in the prediction process. The experimental results show that RWHN obviously exceeds in predicting essential proteins ten other competing methods.

**Conclusions:**

We demonstrated that integrating multi-source data into a heterogeneous network can preserve the complex relationship among multiple biological data and improve the prediction accuracy of essential proteins. RWHN, our proposed method, is effective for the prediction of essential proteins.

## Background

After being removing, the essential protein will cause relevant protein complex losing its function and render the organism being unable to survive or develop. Identifying essential proteins helps us to understand the minimal requirement for cellular survival and development, and plays a vital role in synthetic biology. The study of essential proteins provides valuable information for medicine and other related disciplines, especially in the diagnosis and treatment of diseases, drug design. In biology, essential proteins are primarily identified by biomedical experiments. These methods are expensive, inefficient and time-consuming. Thus, it has become one of the hot issue that proposing efficient computational method for essential proteins identification. Most of calculative methods of essential proteins identification are based on the PPI network. Jeong H et al. [1] proposed the centrality-lethality rule and pointed out that the essentiality of proteins is closely related to the network topology. Inspired by the discovery, several classic network topology-based centrality methods have been developed, such as Degree Centrality (DC) [2], Information Centrality (IC) [3], Closeness Centrality (CC) [4], Betweenness Centrality (BC) [5], Subgraph Centrality (SC) [6] and Neighbor Centrality (NC) [7]. Ning K et al. [8] proposed a measure of centrality based on inverse nearest neighbour of protein networks. Estrada et al. [9] found that less dichotomous proteins were more likely to be essential proteins. Yu et al. [10] discovered the bottleneck node in the network is often the essential proteins. Additionally, the strategy based on node deletion [11] is an effective way to measure the importance of nodes. Most of these methods rarely analyse the intrinsic properties of other known essential proteins, but solely use the topological features of the network. In addition, there is noise in the interaction data due to the restriction of experimental condition, which will affect the accuracy of essential proteins identification. It is urgent to improve fault-tolerance ability of the identification algorithm to the false positive data in PPI networks.

To overcome the limitation of topology-based features, researchers identified essential proteins by combining topological features and other biological information. By combining network topological properties and complex information, Ren J et al. [12] proposed the complex centrality method, named Edge Clustering Coefficient (ECC). Li M et al. [13] combined interaction data and gene expression data to design a method called PeC for predicting essential proteins. As an improved version of the PeC approach, Co-Expression Weighted by Clustering coefficient (CoEWC) [14] was proposed a method of essential protein detection, named, which combined the features of network topology and co-expression property of proteins based on gene expression profile. In our previous work, we proposed an overlapping module mining-based method of essential protein identification, named POEM [15]. In this method, gene expression data and network topology attributes are integrated to construct a reliable weighted network. Combined with homologous information and PPI networks, Peng W et al. [16] proposed an iterative essential protein prediction method, named ION.

In recent years, a variety of methods of essential protein identification has been proposed by integrating multiple biological information. Li M et al. [17] proposed the joint complex centrality by combining the complex information and network topology properties. Luo J et al. [18] adopted the gene expression data, complex information for prediction of essential proteins based on edge aggregation coefficient. Considering the conservation and modularity of essential proteins, we have developed a method named PEMC [19] to identify essential proteins by combining domain information, homologous information and gene expression data. Based on the optimization by artificial fish swarm, the AFSO_EP [20] method was proposed for essential proteins identification, in which the PPI network, gene expression, GO annotation and sub-cellular localization information are integrated to establish a weighted network.

From the above descriptions we can draw a conclusion that existing essential proteins identification approaches aim to improve the predicting accuracy by combining multiple biological data to make up the defects of incomplete PPI data. Such data includes gene expression data, protein domain data, and protein complex data and so on. Generally, they constructed a single network by weighting and summarizing PPI data and multiple biological data, and employed graph-based methods, iterative approaches, and so on to detect essential proteins. However, the way of constructing a reliable single network is easy to ignore the difference of biological feature and functional correlation, coving up intrinsic attributes of heterogeneous data. To overcome the limitation, we construct a heterogeneous network based on the PPI network and protein domains, and proposed a novel computational model called RWHN to predict essential proteins. Firstly, we construct the weighted protein-protein interaction network PN and domain-domain association network DN according to the original PPI network and the known protein-domain association network PDN. And then, we establish a new heterogeneous network by combining the above two constructed networks with the protein-domain association network. Finally, we adopt the improved random walk algorithm to identify essential proteins from the heterogeneous network. To evaluate the performance of newly proposed method, we employ our RWHN, as well as ten state-of-the-art essential proteins prediction methods on two yeast PPI networks and the *E. coli* PPI network. Experimental results demonstrate that our RWTH significantly outperform ten other competitive methods.

## Methods

### Construct weighted protein-protein interaction network *PN*

To reduce the negative impact of false positives, we construct a weighted PPI network according to the analysis of topology of PPI network. The weight of an interaction represents its existence probability or reliability.

For a pair of proteins *p*_*i*_ and *p*_*j*_, we use the improved aggregation coefficient to calculate the weight of interaction between proteins in PPI networks. *WP* is used to represent the relationship between protein pairs. So, the weight of edge (*p*_*i*_, *p*_*j*_) can be defined as:

1 $$ WP\left({p}_i,{p}_j\right)=\left\{\begin{array}{ccc}\frac{{\left|{N}_{p_i}\cap {N}_{p_j}\right|}^2}{\left(|{N}_{p_i}|-1\right)\ast \left(|{N}_{p_j}|-1\right)}&, & if\mid {N}_{p_i}\mid >1\  and\mid {N}_{p_j}\mid >1\\ {}0&, & otherwise\end{array}\right. $$

Where *N*_*pi*_ and *N*_*pj*_ is represented as the list of the direct neighbour nodes of protein *p*_*i*_ and protein *p*_*j*_, respectively, ${N}_{p_i}\cap {N}_{p_j}$ is the common neighbour nodes set of protein *p*_*i*_ and protein *p*_*j*_.

### Construct known protein- domain association network *PDN*

Protein-domain association (*PDN*) is constructed directly based on domain information. If protein *p*_*i*_ contains domain *d*_*j*_, *p*_*i*_ connects domain *d*_*j*_ with a edge in the network *PDN* then *M*_*PD*_ (*i,j*) = 1, otherwise there is no edge between them and *M*_*PD*_ (*i,j*) = 0. *M*_*PD*_ is the adjacency matrix corresponding to the network *PDN*.

### Construct domain-domain association network *DN*

Research [21] has verified the high correlation between protein domains and the essentiality of proteins. Motivated by it, protein domains data is adopted when establishing the heterogeneous network. The domain-domain association network *DN* is constructed on the basis of the above constructed *PN* network and the known protein-domain association network *PDN*. Let *d*_*i*_ and *d*_*j*_ be two different domains, we select the maximum from *WP*(*p*_*x*_*, p*_*y*_) as the association between a given protein *p*_*y*_ and protein group *P*(*d*_*j*_), which can be calculated as follows:

2 $$ S\left({p}_y,P\left({d}_j\right)\right)=\underset{p_x\in P\left({d}_j\right)}{\max}\left( WP\left({p}_x,{p}_y\right)\right) $$

Based on Eq. (2), for each pair of domain *d*_*i*_ and domain *d*_*j*_, the weight between them can be calculated as follows:

3 $$ WD\left({d}_i,{d}_j\right)=\frac{\sum_{p_y\in P\left({d}_i\right)}S\left({p}_y,P\left({d}_j\right)\right)+{\sum}_{p_x\in P\left({d}_j\right)}S\left({p}_x,P\left({d}_i\right)\right)}{\mid P\left({d}_i\right)\mid +\mid P\left({d}_j\right)\mid } $$

Where *P*(*d*_*i*_) and *P*(*d*_*j*_) are represented the protein set of domain *d*_*i*_ and domain *d*_*j*_, respectively and *S*(*p*_*y*_, *P*(*d*_*j*_)) denotes the association between protein *p*_*y*_ and the set of protein *P*(*d*_*j*_).

### Initializing the score vector of proteins and domains

In this paper, the functional feature derived from subcellular localization information and conservative feature obtained by homologous information are both taken into account when scoring proteins. Firstly, we calculate the important score of subcellular localization, which can be expressed as:

4 $$ Sub(i)=\frac{\mid P(i)\mid }{\underset{1\le j\le m}{\max}\left(|P(j)|\right)} $$

Where |*P*(*i*)| is the number of proteins associated with *i*-th subcellular localization, *m* is the total number of different types of subcellular localization. For a given protein *p*_*i*_, its functional score can be computed as follows:

5 $$ S\_ Score\left({p}_i\right)=\underset{j\in S\left({p}_i\right)}{\max}\left( Sub(j)\right) $$

Where *S*(*p*_*i*_) is a list of subcellular location list associated with the protein *p*_*i*_.

The conservative score for the protein *p*_*i*_ is obtained from homologous information and defined as follow:

6 $$ I\_ Score\left({p}_i\right)=\frac{I\left({p}_i\right)}{\underset{1\le j\le n}{\max}\left(I\left({p}_j\right)\right)} $$

After getting the functional score and the conservative score of a protein, its initial score is defined as:

7 $$ {h}_0\left({p}_i\right)=\left(S\_ Score\left({p}_i\right)+I\_ Score\left({p}_i\right)\right)/2 $$

As for domains, their initial scores are derived from scores of their relevant proteins. Given a domain *d*_*j*_, its initializing score is computed by using the following formula:

8 $$ {h}_0\left({d}_j\right)=\underset{p_x\in S\_P\left({d}_j\right)}{\max}\left({h}_0\left({p}_x\right)\right) $$

Where *S_P*(*d*_*j*_) is a list of proteins that contain the domain *d*_*j*_.

### Random walk for the heterogeneous network

According to the three constructed network *PN*, *PDN* and *DN*, our prediction model RWHN based on random walk consists of the following three steps:

- Step 1: Establishing the heterogeneous matrix *HM*

Networks *PN*, *DN* and *PDN* can be represent as the *n* × *n* adjacency matrix *M*_*P*_, *m* × *m* adjacency matrix *M*_*D*_ and *n* × *m* adjacency matrix *M*_*PD*_, respectively, in which *n* and *m* denotes the number of proteins and domains separately. Thus, a heterogeneous matrix *HM* is constructed and formally expressed as follows:

9 $$ HM=\left[\begin{array}{cc}{M}_P& {M}_{PD}\\ {}{M^T}_{PD}& {M}_D\end{array}\right] $$

**Fig. 1:**
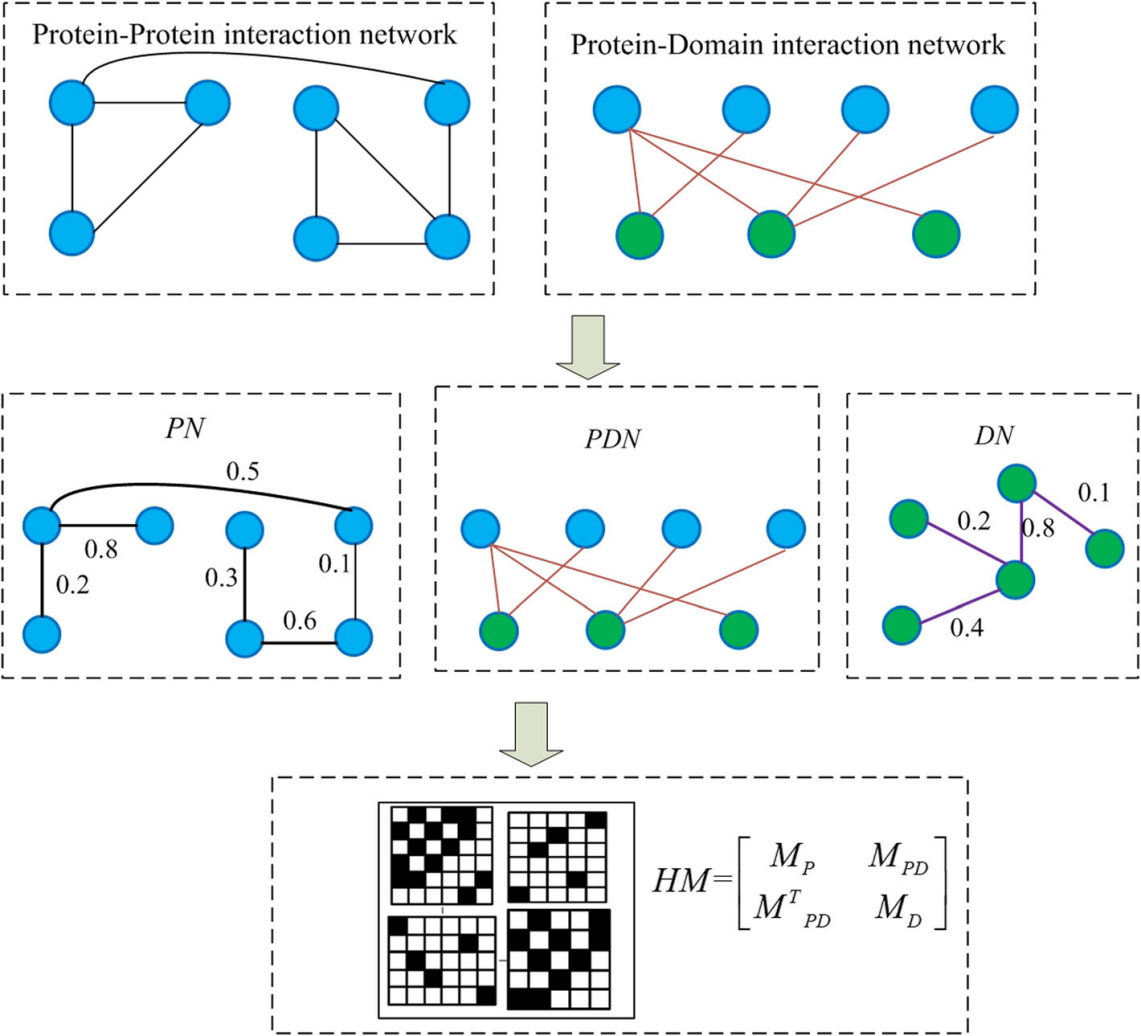
Schematic diagram of the heterogeneous matrix construction. This figure shows how to construct a heterogeneous matrix. The input files include original protein-protein interaction network and protein domain information. Blue nodes and red nodes represent proteins and domains, respectively

Where, *M*^*T*^_*PD*_ is a transport matrix of the matrix *M*_*PD*_. Figure 1 illustrates the process of establishing the heterogeneous matrix *HM*.

- Step 2: Establishing the transition probability matrix *HM_P* as follow:

In this work, we construct the transition probability matrix *HM_P* by normalized operation, which is calculated as follow:

10 $$ HM\_P=\left[\begin{array}{cc}{PM}_P& {PM}_{PD}\\ {}{PM^T}_{PD}& {PM}_D\end{array}\right] $$

The transition probability from protein *p*_*i*_ to protein *p*_*j*_ is defined as:

11 $$ {PM}_p\left(i,j\right)=p\left({p}_j|{p}_i\right)=\left\{\begin{array}{ccc} WP\left(i,j\right)/{\sum}_j WP\left(i,j\right)&, & if{\sum}_j{M}_{PD}\left(i,j\right)=0\\ {}\left(1-\beta \right) WP\left(i,j\right)/{\sum}_j WP\left(i,j\right)&, & otherwise\end{array}\right. $$

The transition probability from domain *d*_*i*_ to domain *d*_*j*_ is defined as:

12 $$ {PM}_d\left(i,j\right)=p\left({d}_j|{d}_i\right)=\left\{\begin{array}{ccc} WD\left(i,j\right)/{\sum}_j WD\left(i,j\right)&, & if{\sum}_j{M}_{PD}\left(j,i\right)=0\\ {}\left(1-\beta \right) WD\left(i,j\right)/{\sum}_j WD\left(i,j\right)&, & otherwise\end{array}\right. $$

The transition probability from protein *p*_*i*_ to domain *d*_*j*_ is defined as:

13 $$ {PM}_p\left(i,j\right)=p\left({d}_j|{p}_i\right)=\left\{\begin{array}{ccc}\beta {M}_{PD}\left(i,j\right)/{\sum}_j{M}_{PD}\left(i,j\right)&, & if{\sum}_j{M}_{PD}\left(i,j\right)\ne 0\\ {}0&, & otherwise\end{array}\right. $$

The transition probability from protein *p*_*i*_ to protein *p*_*j*_ is defined as:

14 $$ {PM}_p\left(i,j\right)=p\left({p}_j|{d}_i\right)=\left\{\begin{array}{ccc}\beta {M}_{PD}\left(j,i\right)/{\sum}_j{M}_{PD}\left(j,i\right)&, & if{\sum}_j{M}_{PD}\left(j,i\right)\ne 0\\ {}0&, & otherwise\end{array}\right. $$

The parameter *β* denotes the moving probability of the movement from the weighted protein-protein interaction network *PN* to the domain-domain association network *DN*.

- Step 3: Randomly walking in the heterogeneous based on the PageRank algorithm.

In this paper, we employ the RageRank algorithm in the transition probability matrix *HM_P* to iteratively score proteins. Assumed that the walker arrive at the current position after experiencing *i*-th step. Then we can update the walk probability vector *h*_(*i* + 1)_ for each node (proteins and domains) in the heterogeneous network according to the transition probability matrix *HM_P*. For sake of calculating the score vector *h* of protein and domain, we use the equation as follow:

15 $$ {h}_{i+1}=\left(1-\alpha \right) HM\_{Ph}_i+\alpha {h}_0 $$

The parameter *α* is used to adjust the proportion of initial score and last iteration score and *h*_0_ is jump probability. The overall framework of newly proposed prediction model RWHN can be illustrated as the Algorithm 1.

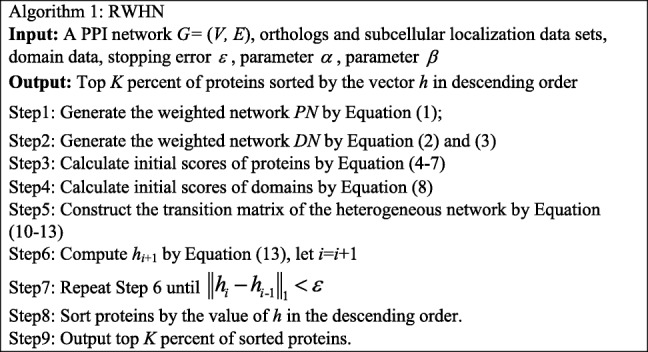



## Results

### Experimental data

For evaluation of the prediction performance of RWHN, we implemented our method and other ten state-of-the-art methods: Degree Centrality (DC) [2], Information Centrality (IC) [3], Closeness Centrality (CC) [4], Betweenness Centrality (BC) [5], Subgraph Centrality (SC) [6], Neighbor Centrality (NC) [7], PeC [13], CoEWC [14], POEM [15] and ION [16]) on prediction of essential genes by using two *Saccharomyces cerevisiae* (yeast) PPI networks: DIP dataset [22] and Gavin dataset [23]. We will represent the experimental results on DIP data set in detail and the result on Gavin dataset briefly. In both DIP and Gavin dataset, self-interactions and repeated interactions are filtered out. There are 5093 proteins and 24,743 interactions in DIP dataset. The Gavin dataset consists of 1855 proteins and 7669 interactions. As the basis of the heterogeneous network, the domain data is downloaded from Pfam database [24]. There are 1081 and 744 different types of domains contained in the DIP and Gavin dataset, respectively. So, the heterogeneous matrix *HM* derived from DIP and Gavin is (5093 + 1081) × (5093 + 1081) and (1855 + 744) × (1855 + 744) separately.

The subcellular localization information of proteins used for scoring protein is derived from COMPARTMENTS database [25] (Downloaded on Apr 20th 2014). In this paper, we only reserve 11 categories subcellular localizations (or compartments) closely related to essential proteins in a eukaryotic cell of COMPARTMENTS database: Endoplasmic, Cytoskeleton, Golgi, Cytosol, Vacuole, Mitochondrion, Endosome, Plasma, Nucleus, Peroxisome and Extracellular. Information on orthologous proteins also used to initial score vectors of proteins and domains comes from the InParanoid database (Version 7) [26], which involving a collection of pair wise comparisons between 100 whole genomes.

A benchmark set of essential genes of *Saccharomyces cerevisiae* consisting 1285 essential genes, are derived from the following four databases: MIPS [27], SGD [28], DEG [29], and SGDP [30]. Among all 5093 proteins in the DIP network, 1167 proteins are essential and 3526 proteins are non-essential. There are 714 true essential proteins among 1855 proteins in the Gavin PPI network.

### Comparison with ten essential proteins prediction methods

**Fig. 2:**
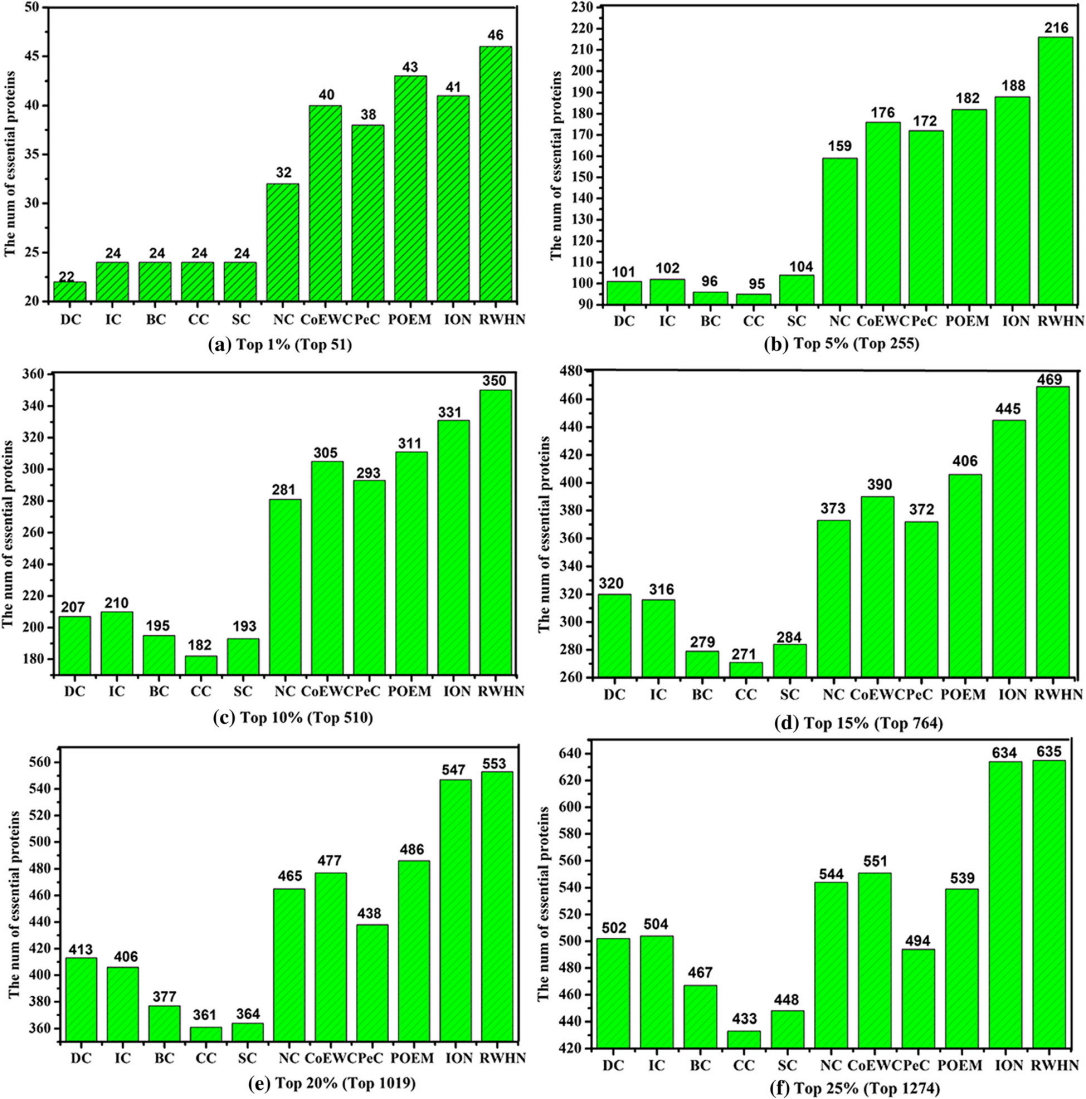
**a** Top 1% ranked proteins. **b** Top 5% ranked proteins. **c** Top 10% ranked proteins. **d** Top 15% ranked proteins. **e** Top 20% ranked proteins. **f** Top 25% ranked proteins. Comparison of the number of essential proteins predicted by RWHN and ten other competitive methods. The proteins in PPI network are ranked in the descending order based on their ranking scores computed by RWHN, Degree Centrality (DC), Information Centrality (IC), Closeness Centrality (CC), Betweenness Centrality (BC), Subgraph Centrality (SC), Neighbor Centrality (NC), PeC, CoEWC, POEM and ION. Then, top 1, 5, 10, 15, 20 and 25% of the ranked proteins are selected as candidates for essential proteins. According to the list of known essential proteins, the number of true essential proteins is used to judge the performance of each method. The figure shows the number of true essential proteins identified by each method in each top percentage of ranked proteins. Since the total number of ranked proteins is 5093. The digits in brackets denote the number of proteins ranked in each top percentage

To evaluate the performance of newly proposed essential proteins prediction method, RWHN, we compare the number of essential proteins identified by RWHN (*α* =0.3, *β* =0.2) and ten other competing essential proteins prediction methods, when picking out various top percentages of ranked proteins as candidates for essential proteins. Figure 2 indicates the comparison results between RWHN and ten methods.

As shown in Fig. 2, the prediction performance of RWHN significantly outperforms other ten competitive methods for the identification of essential proteins. With top 1% of proteins selected, RWHN obtains a prediction accurary of 90.19%. By selecting top 5% of protiens, RWHN can detect 84.70% of true essential proteins. For top 10% of selected proteins, RWHN is capable of acquiring prediction accurary of 68.62%, which is 92.31% higher than CC. In addition, Compared with NC which has the best performance among six network topology-based methods (DC, IC, BC, CC, SC and NC), in each top percentage, the prediction accuracy of RWHN is respectively improved by 43.75, 35.85, 24.56, 25.74, 18.92 and 16.73%. Especially, in top 1% of ranked proteins, RWHN is able to identify twice or more as many essential proteins as DC. Unfortunately, with more candicate proteins selected, the advantage of RWHN in the prediction of essential proteins becomes growing slowly. However, compared with CoEWC, PeC, POEM and ION, which detect essential proteins by integrating PPI networks topolgy and muitiple biological data, our RWHN also outperforms these four methods. From Fig. 2, we can draw a conclusion that RWHN always gets the highest prediction accurary from top 1% to top 25%.

### Validation with jackknife methodology

**Fig. 3:**
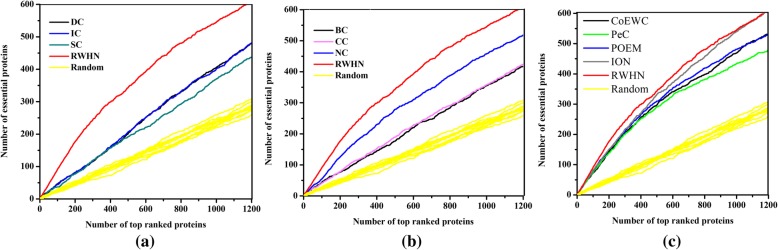
Jackknife curves of RWHN and ten other existing centrality methods. The x-axis represents the proteins in PPI network ranked by RWHN and ten other methods, ranked from left to right as strongest to weakest prediction of essentiality. The Y-axis is the cumulative count of essential proteins encountered moving left to right through the ranked. The areas under the curve for RWHN and the ten other methods are used to compare their prediction performance. In addition, the 10 random assortments are also plotted for comparison. **a** shows the comparison results of RWHN, DC, IC, SC and DC. **b** shows the comparison results of RWHN, BC, CC and NC. **c** shows the comparison results of RWHN and other four methods: PeC, CoEWC, POEM and ION

For overall comparison, the jackknife methodology [31] is used to examine the prediction performance of RWHN and the ten other existing centrality methods. The experimental results are described in Fig. 3. In Fig. 3, the *X*-axis represents identified essential proteins of the descending order in PPI networks from the left to the right. This order is according to their ranking scores calculated by their corresponding method. And the *Y*-axis means the cumulative count of true essential proteins with respect to ranked proteins of all methods. The areas under the curve (AUC) for RWHN and ten other existing essential protein prediction methods are used to compare their prediction performance. What is more, the 10 random assortments are also plotted for comparison. Figure 3a shows the comparison result of RWHN and three centrality methods: DC, IC and SC. From this figure we can see that, RWHN has consistently excelled these three methods. Figure 3b illustrates the comparison result of RWHN and three other centrality methods: BC, CC and NC. RWHN still surpasses that of any other method in terms of prediction accuracy among these methods. Figure 3c shows the comparison result of RWHN and other four multiple biological data integrated methods: CoEWC, PeC, POEM and ION. From Fig. 3, we can see that the performance gap becomes small between RWHN and these four essential proteins identification methods. And when the number of ranked proteins comes near to 1200, the curve of RWHN and the curve of ION almost overlap. Even so, RWHN still gets the better of CoEWC, PeC, POEM and ION. Furthermore, all of these eleven methods achieve better prediction performance than the randomized sorting.

### Analysis of the differences between RWHN and the ten method

In order to analyze why and how RWHN gets better results than the ten other competitive centrality methods, we compare identified proteins ranked top 200 by each method (DC, IC, SC, BC, CC, NC, PeC, CoEWC, POEM, ION and RWHN). The results of the comparison are to view how many common and different proteins are identified by these methods. It is shown in following table that the number of overlaps and different proteins between RWHN and any of ten other competitive essential proteins detection methods. |RWHN∩Mi| denotes the number of overlaps proteins detected by both RWHN and one of the ten other existing prediction methods Mi. {Mi − RWHN} represents the list of proteins detected by Mi ignored by RWHN. |Mi − RWHN| is the number of proteins in set {Mi − RWHN}.

As shown in the Table 1, among the top 200 proteins, there exist wide difference between the proteins discovered by both RWHN and other ten competing prediction methods. From the second column of Table 1, we can see that the proportion of overlapping proteins detected by RWHN and DC, IC, SC, BC, CC are all less than 15%, which means there are almost no overlapping proteins identified by RWHN and them. For NC, the proportion of overlapping proteins predicted by RWHN and NC are not more than 25%. There are only few overlapping protiens predicted by RWHN and NC. Besides, the proportion of overlapping proteins predicted by RWHN and PeC, CoEWC, POEM are less than 35% and the proportion of overlapping proteins identified by RWHN and ION is 55%. There are more than 40% of these different proteins are non-essential proteins. The maximun proportion of non-essential proteins is up to 68%. Additionally, according to these non-essential proteins predicted by other methods, we can find that more than 70% of non-essential proteins in top 200 possess quite low ranking scores computed by RWHN. For example, we also can see that about 89% of non-essential proteins among the top 200 of proteins predicted by BC or CC have been marked low scores in RWHN. Moreover, there are also about 70% of non-essential proteins in the result of the POEM method with low RWHN scores. This implies that RWHN can reject a lot of non-essential proteins which can not be overlook by other prediction methods. The results indicates that RWHN is a special and effective method comapred with ten other competing essential proteins prediction methods.

**Table 1:** Common and different genes predicted by RWHN and other competing methods ranked in top 200 proteins

Centrality measures (Mi)	|RWHN∩Mi|	|Mi − RWHN |	Non-essential proteins in {Mi − RWHN}	Percentage of non-essential proteins in {Mi − RWHN} with low RWHN value
DC	27	173	118	83.90%
IC	26	174	118	84.75%
SC	24	176	120	87.50%
BC	23	177	118	89.83%
CC	23	177	110	89.09%
NC	44	156	73	86.30%
PeC	68	132	53	79.25%
CoEWC	69	131	51	76.47%
POEM	69	131	46	71.74%
ION	110	90	40	82.50%

This table shows the common and the difference between RWHN and the ten other competing methods (DC, IC, SC, BC, CC, NC, PeC, CoEWC, POEM and ION) when predicting top 200 proteins. |RWHN∩Mi | denotes the number of proteins identified by both RWHN and one of the ten other methods Mi. {Mi − RWHN} represents the set of proteins detected by Mi while ignored by RWHN. |Mi − RWHN| is the number of proteins in set {Mi − RWHN}. The last column describes the percentages of different nonessential proteins with low RWHN scores (less than 0.2) in top 200 proteins

**Fig. 4:**
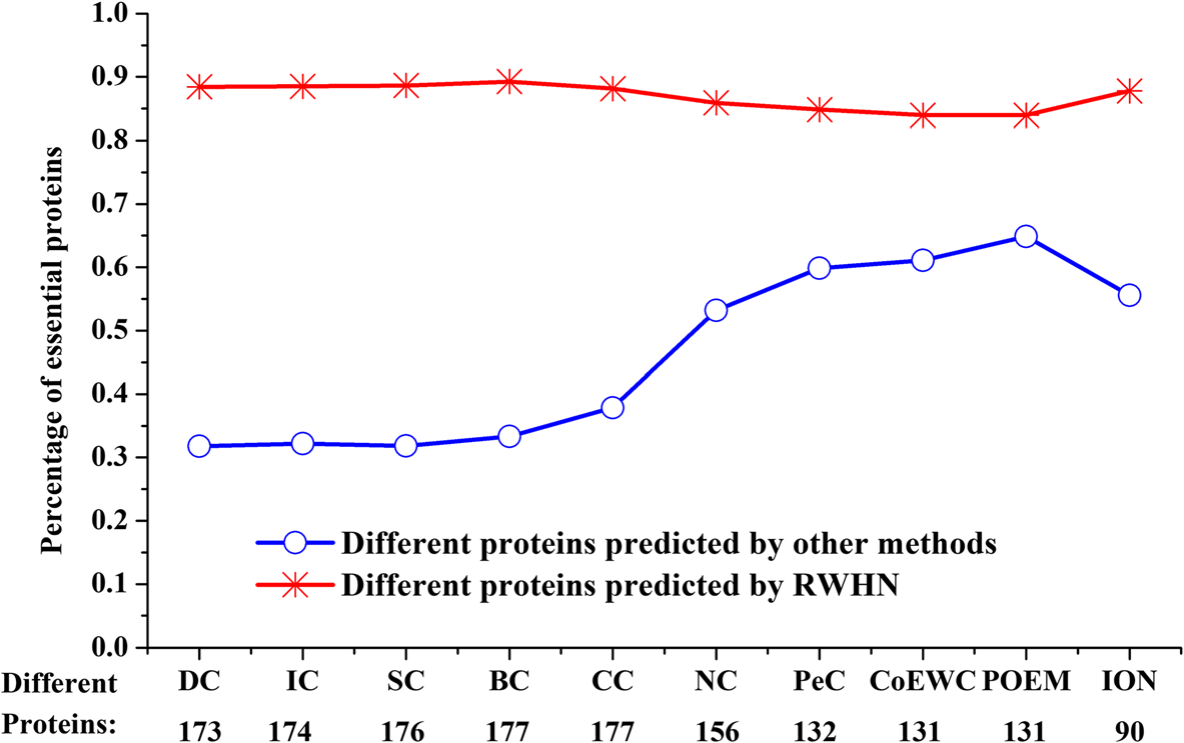
Percentages of different essential proteins predicted by RWHN and ten other competing prediction methods. Different proteins between two prediction methods are the proteins predicted by one method while neglected by the other method. The figure shows the percentages of the essential proteins in the different proteins between RWHN and ten other competing methods (DC, IC, SC, BC, CC, NC, PeC, CoEWC, POEM and ION), respectively

For further comparsion, we make a statistical analysis the percentages of different essential protiens detected by RWHN and these competitive methods. Figure 4 shows the percentage of essential proteins all of different proteins between RWHN and ten other competing prediction methods. As illustrated in Fig. 4, RWHN always can identify more different essential proteins than other methods. Compared with POEM, there are 131 different proteins detected by RWHN. About 86% of these proteins are essential. On the contrary, there are only 64.88% of different proteins detected by POEM while overlooked by RWHN are essential proteins. In fact, among the top 200 of proteins, RWHN can discover more different essential proteins which can not be predicted by anyone of the ten other essential proteins identification methods. From the above, RWHN can not only detect more essential proteins ignored by ten other competing prediction methods but also reject a mass of non-essential proteins which can not be overlooked by these methods. These statistical results are not difficult to explain why the RWHN method can achieve high essential proteins prediction performance.

### Validated by precision-recall curves

**Fig. 5:**
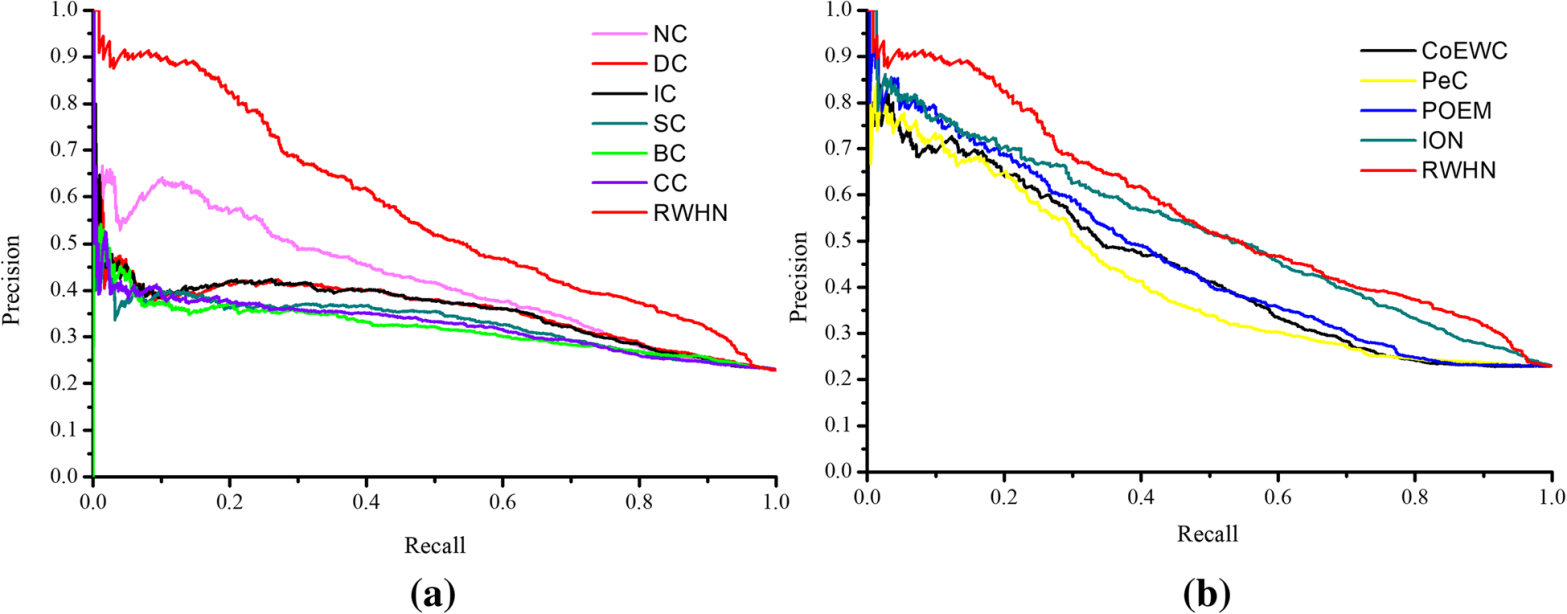
PR curves of RWHN and ten other existing centrality methods. The proteins ranked in top K (cut-off value) by each method (RWHN, DC, IC, SC, BC, CC, NC, PeC, CoEWC, POEM and ION) are selected as candidate essential proteins (positive data set) and the remaining proteins in PPI network are regarded as candidate nonessential proteins (negative data set). With different values of K selected, the values of precision and recall are computed for each method. The values of precision and recall are plotted in PR curves with different cut-off values. **a** shows the PR curves of RWHN, DC, IC, SC, BC, CC and NC. **b** shows the PR curves of RWHN and other four methods: CoEWC, PeC, POEM and ION

Moreover, the precision-recall (PR) curve is adopted to evaluate the overall performance of RWHN, as well as other ten methods. Firstly, the proteins in PPI networks are ranked in descending order based on scores obtained from each method. After that, top K proteins are picked out and put into the positive set (candidate essential genes), the rest of proteins in PPI networks are stored in the negative set (candidate non-essential genes). The cut-off parameter of K went from 1 to 5093. With different values of K picked out, the values of precision and recall are calculated by each approach, respectively. Finally, the PR curves are plotted according to values of precision and recall when K changes in the interval [1, 5093]. Figure 5a shows the PR curves of RWHN and six topology-based centrality methods: DC, IC, BC, CC, SC and NC. Figure 5b illustrates the PR curves of RWHN, as well as other four methods: PeC, CoEWC, POEM and ION. Figure 5 indicates that the PR of RWHN is clearly above those of all competitive centrality methods.

### Effects of parameters α and β

**Fig. 6:**
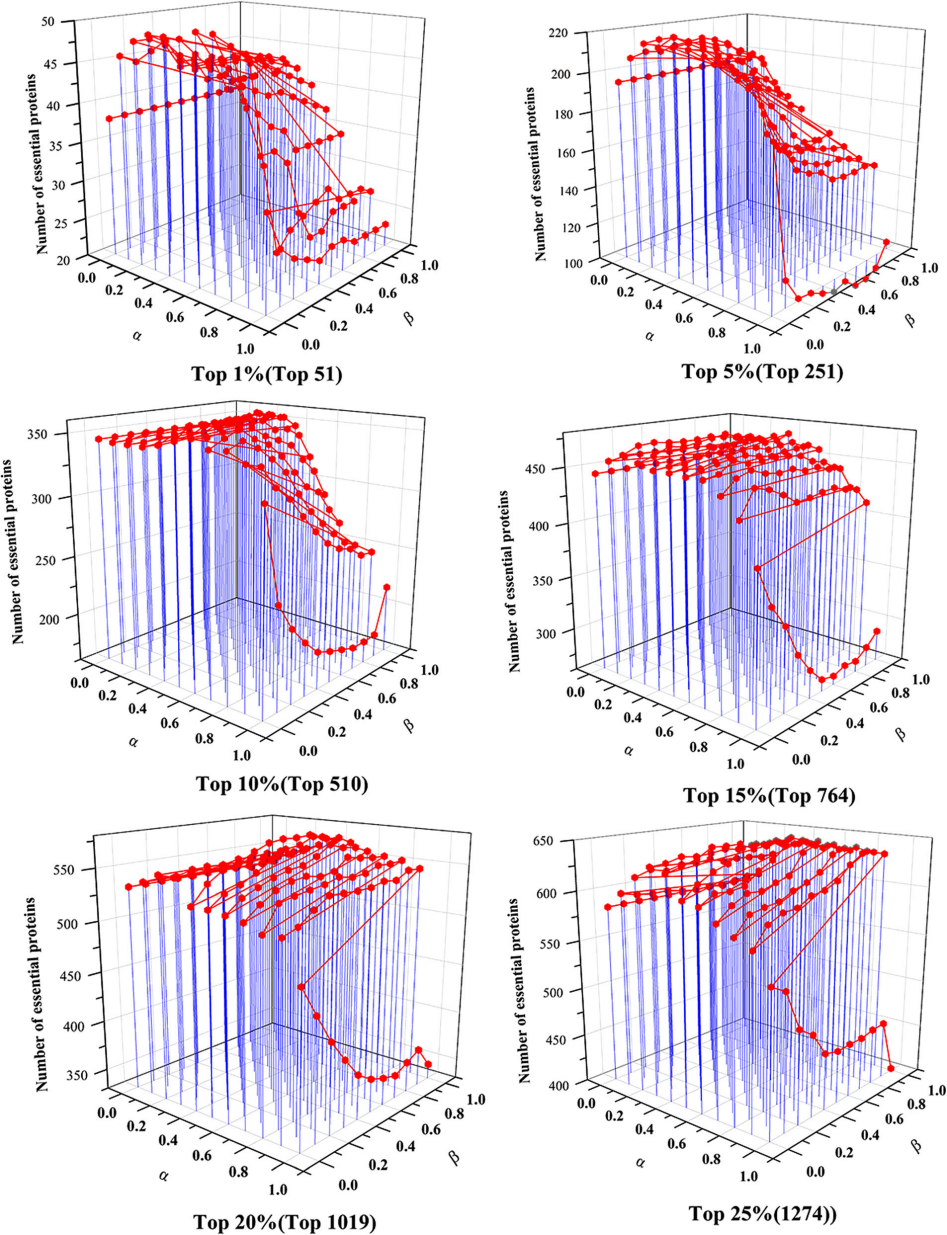
The analysis of parameters α and β. The figure shows the effect of parameter α and β on the performance of RWHN. Six figures represents prediction accuracy of RWHN in each top percentage of ranked proteins by setting different values of α and β, ranging from 0 to 1

In RWHN, we employ two self-defined parameters α and β. α is used to adjust the proportion of the functional score and the conservative score for initial scores of proteins. The parameter β represents the moving probability from the weighted protein-protein interaction network *PN* to the domain-domain association network *DN*. For evaluating the effects of these two parameters on prediction performance of RWHN, we set different values of α and β ranging from 0 to 1. Figure 6 shows the detailed results with the two parameters changing in RWHN. Here, we pick out from top 1% to top 25% proteins identified by RWHN. The prediction accuracy is evaluated according to the number of true essential proteins as candidates. When the value of α is 0.6 or 0.7 and β is set as 0, among top 1% proteins selected, the true essential proteins are up to 50 identified by RWHN and the prediction accuracy is near 100%, but the accuracy is declining in the top 5% to top 25% of proteins selected. On the whole, the closer α value is to 1, the lower the prediction accuracy is. In addition, when α is set as 0.3 and β is arbitrarily assigned between 0 and 1, the average number of true essential proteins predicted from top 1 to 25% is 45, 202, 351, 467, 553, and 634, respectively. And when α is equal to 0.3 and β is set as 0.2, the number of true essential proteins is closest to the average. As a result, we think the optimum α and β on the DIP dataset is 0.3, 0.2, respectively. As for the Gavin dataset, the optimum α and β is 0.3, 0.1, respectively.

### Prediction performance of RWHN based on Gavin dataset

**Fig. 7:**
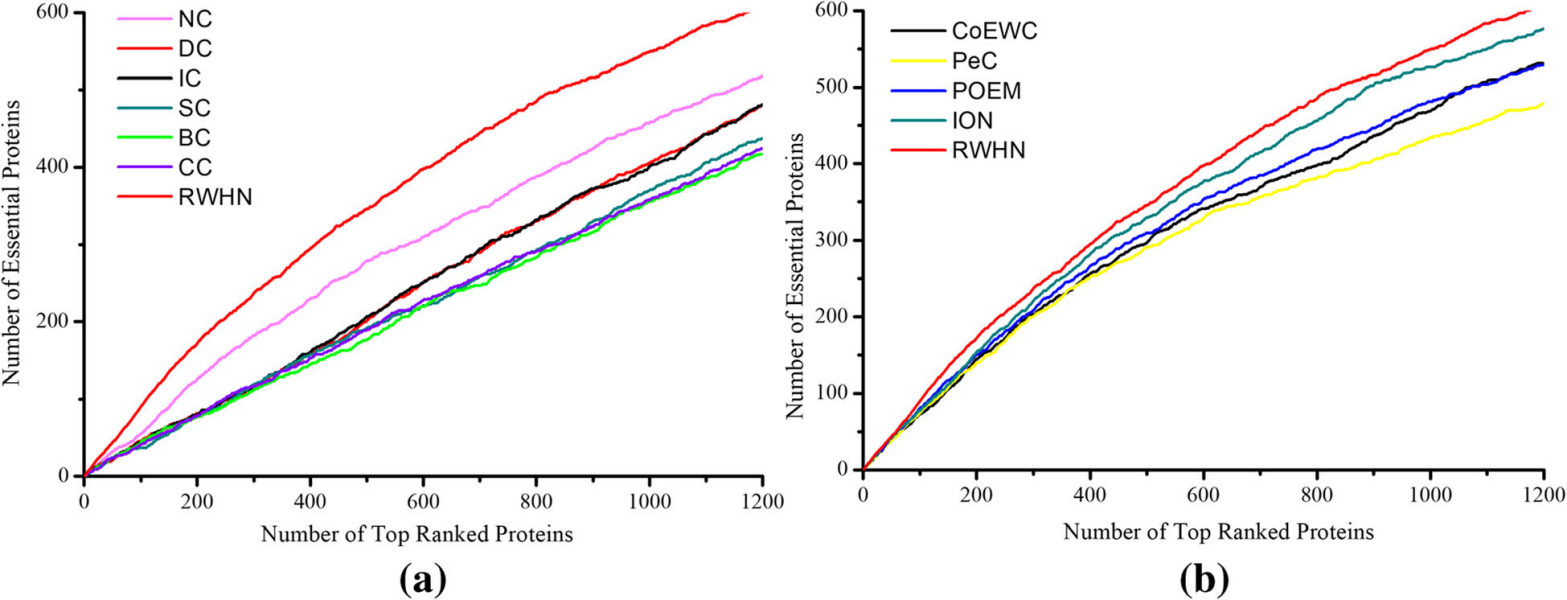
**a** Shows the comparison results of RWHN, DC, IC, BC, CC, SC and NC. **b** Shows the comparison results of RWHN, CoEWC, PeC, POEM and ION. Comparison results by a jackknife methodology using Gavin dataset. The prediction performance of RWHN and ten other competing methods (DC, IC, SC, BC, CC, NC, PeC, CoEWC, POEM and ION) based on the Gavin dataset are validated by the jackknife methodology

To further test the performance of RWHN, we perform the prediction of essential proteins based on PPI data from Gavin dataset. Table 2 shows the comparison of the number of essential proteins identified by RWHN and ten other essential proteins prediction methods. From Table 2, we can see that the prediction accuracy of RWHN among top 1% and top 5% proteins are more than 89%. From top 1% to top 25% predicted proteins, the RWHN method still outperforms ten other competing prediction methods in the Gavin dataset. The jackknife curves of each method and the 10 random assortments are illustrated in Fig. 7. All of these experimental results show that RWHN has better performance in predicting essential proteins than the ten other competitive methods on Gavin dataset.

**Table 2:** Number of essential proteins predicted by RWHN and ten competing methods based on the Gavin dataset

Methods	1%(19)	5%(93)	10%(196)	15%(279)	20%(371)	25%(464)
DC	12	44	80	106	145	182
IC	11	42	79	108	147	189
SC	9	36	77	109	146	179
BC	10	40	76	103	134	163
CC	9	38	77	113	141	175
NC	11	51	123	170	213	259
PeC	15	69	142	193	238	285
CoEWC	16	69	136	190	237	275
POEM	17	74	148	199	249	296
ION	17	73	150	207	263	312
RWHN	18	83	169	222	277	330

This table shows the comparison of the number of essential proteins identified by RWHN and ten other competing methods (DC, IC, SC, BC, CC, NC, PeC, CoEWC, POEM and ION) based on the Gavin dataset. The total number of ranked proteins in Gavin dataset is 1855. The digits in brackets denote the number of proteins ranked in each top percentage

### Prediction performance of RWHN based on protein data from *E. coli*

**Fig. 8:**
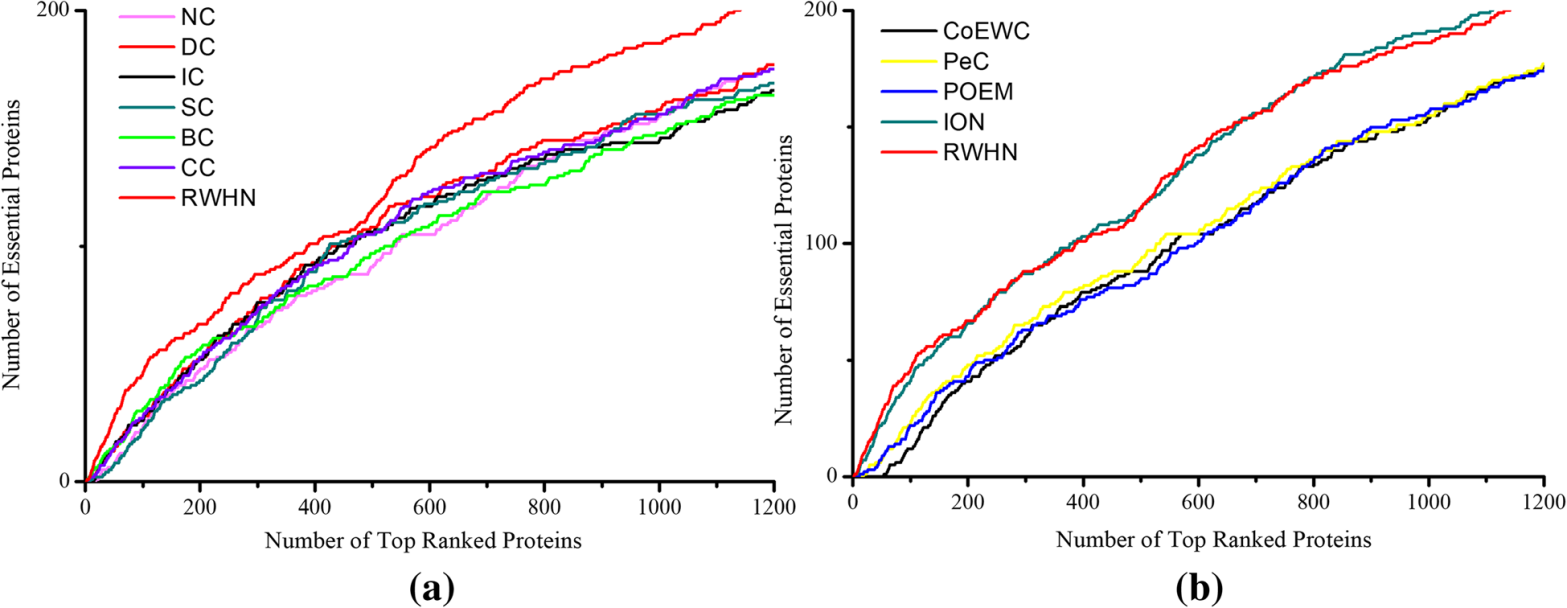
**a** Shows the PR curves of RWHN and six network topology-based methods: DC, IC, BC, CC, SC and NC. **b** Shows the PR curves of RWHN and other four methods: PeC, CoEWC, POEM and ION. Comparison results by a jackknife methodology on protein data from *E. coli.* The prediction performance of RWHN and ten other competing methods (DC, IC, SC, BC, CC, NC, PeC, CoEWC, POEM and ION) based on protein data from *E. coli* are validated by the jackknife methodology

Moreover, we run our RWHN and other competing methods on the species of *E. coli*. The PPI network of *E. coli* is also downloaded from DIP database, which consists of 2727 proteins and 11,803 interactions. Among these 2727 proteins, there are 254 essential proteins and 2474 non-essential proteins. The proportion of essential proteins on *E. coli* (254/2727 = 9.31%) is much smaller than that of yeast (DIP: 1167/5093 = 22.915, Gavin: 714/1855 = 38.49%). The ranking scores of *E. coli* proteins are calculated by using of RWHN (α = 0.2, β = 0.1) and the other competing methods, respectively. The number of essential proteins predicted by eleven methods in top 1%(27), 5%(136), 10%(273), 15%(409), 20%(545) and 25%(682) are list in Table 3. Figure 8 is the jackknife curves of each method. Compared to the results in yeast PPI networks, the prediction accuracy of all these methods decreased obviously, due to the incomplete and inconvincible experimental data. For example, the gene expression profile of *E. coli* only contains 246 proteins, which result in the sharply decline of the performance of PeC, CoEWC and POEM. On the other hand, the PPI network from *E. coli* is sparser than the yeast networks. Even so, our RWHN method still get higher prediction accuracy than DC, IC, SC, BC, CC, NC, PeC, CoEWC and POEM, and comparable results with ION. Specially, as selecting top 1% ranked proteins, RWHN archives 87.50, 114.29, 650, 66.67, 114.29, 400, 400, 400 and 50% improvement than DC, IC, SC, BC, CC, NC, PeC, POEM and ION, respectively.

**Table 3:** Number of essential proteins predicted by RWHN and ten competing methods based on the protein data from *E. coli*

Methods	1%(27)	5%(136)	10%(273)	15%(409)	20%(545)	25%(682)
DC	8	37	69	94	118	129
IC	7	36	68	95	112	127
SC	2	34	60	93	110	124
BC	9	40	65	84	103	120
CC	7	36	67	92	113	130
NC	3	35	60	82	102	118
PeC	3	35	61	82	98	118
CoEWC	0	6	16	24	42	63
POEM	3	32	56	77	92	113
ION	10	52	82	103	125	153
RWHN	15	56	83	103	129	154

This table shows the comparison of the number of essential proteins identified by RWHN and ten other competing methods (DC, IC, SC, BC, CC, NC, PeC, CoEWC, POEM and ION) based on protein data from *E. coli*. The total number of ranked proteins in *E. coli* is 2727. The digits in brackets denote the number of proteins ranked in each top percentage

## Discussions

Essential proteins play a vital role in synthetic biology, the diagnosis and treatment of diseases, drug design, and help us to understand the minimal requirement for cellular survival and development. Computational methods instead of biomedical experiments have been proposed to predict essential proteins from PPI networks. However, PPI data obtained from high throughput technique contains false positives and false negatives. More and more researchers focus on integrating PPI networks and multiple biological data. Here we design a new framework to identify essential proteins by establishing heterogeneous networks based on PPI network topological characteristics and protein domains information. And then, we apply an improved random walk algorithm on the heterogeneous network to calculate the importance scores for candidate essential proteins. These new insights provide good starting points for multiple biological information fusion.

## Conclusions

In this paper, we propose a new essential proteins prediction model named RWHN by combining PPI networks with protein domains, the subcellular localization information and orthologous information. Different from current multiple biological data fusion based methods, we establish a heterogeneous network through integrating the weighted PPI network, domain-domain association network and known protein-domain association network. And then, based on the newly constructed heterogeneous network, a random walk algorithm is adopted to identify essential proteins. Moreover, the functional property and conservative property of essential proteins are both taken into account. Experimental comparison results between RWHN and ten state-of-the-art methods on two yeast PPI networks and the *E. coli* PPI network shows that RWHN significantly outperforms other competing methods. The results also indicate that RWHN is a special and effective method for essential proteins prediction.

## Data Availability

The datasets used and/or analyzed during the current study are available from the first author or corresponding author on reasonable request.

## Abbreviations

BC: Betweenness Centrality
CC: Closeness Centrality
CoEWC: Co-Expression Weighted by Clustering coefficient
DC: Degree Centrality
ECC: Edge Clustering Coefficient
IC: Information Centrality
NC: Neighbor Centrality
PPI: Protein-Protein Interaction
RWHN: Randomly Walking in the Heterogeneous Network
SC: Subgraph Centrality
